# Efficacy of Chinese herbal medicine in the treatment of anxiety and depression in male sexual dysfunction: a systematic review and meta-analysis

**DOI:** 10.1093/sexmed/qfaf048

**Published:** 2025-07-10

**Authors:** Zhaozhan Xie, Jinxian Lu, Xuecheng Zhang, Hongling Jia, Yongchen Zhang

**Affiliations:** School of Acupuncture-Moxibustion and Tuina, Shandong University of Traditional Chinese Medicine, Jinan, Shandong Province 250355, China; School of Acupuncture-Moxibustion and Tuina, Shandong University of Traditional Chinese Medicine, Jinan, Shandong Province 250355, China; China-Japan Friendship Hospital, Chaoyang District, Beijing 100029, China; The Second Affiliated Hospital of Shandong University of Traditional Chinese Medicine, Jinan City, Shandong Province 250001, China; School of Acupuncture-Moxibustion and Tuina, Shandong University of Traditional Chinese Medicine, Jinan, Shandong Province 250355, China

**Keywords:** male sexual dysfunction, Chinese herbal medicine, anxiety, depression, systematic review, psychosexual health

## Abstract

**Background:**

Male Sexual Dysfunction (MSD), comprising erectile dysfunction (ED) and premature ejaculation (PE), exhibits an age-related prevalence affecting 50% of males beyond their fourth decade. Beyond physiological manifestations, MSD with comorbid anxiety and depression exerts profound psychosocial impacts. Emerging evidence suggests Chinese Herbal Medicine (CHM) may offer therapeutic potential for addressing this clinical intersection.

**Aim:**

To systematically assess the efficacy of CHM on alleviating anxiety and depression in patients with MSD via a comprehensive systematic review and meta-analysis.

**Methods:**

This study systematically searched four Chinese databases (China National Knowledge Infrastructure, Wanfang Database, China Biomedical Database, and VIP Database) and four international databases (PubMed, Web of Science, EMBASE, and Cochrane Library). Randomized controlled trials (RCTs) investigating CHM interventions for MSD with comorbid anxiety and depression were identified.

**Outcomes:**

The primary outcome focused on changes in symptoms of anxiety and depression, while secondary outcomes encompassed overall male sexual function improvement.

**Results:**

By synthesizing data from 12 RCTs involving 1050 participants, our findings provide the first robust evidence that CHM significantly alleviates anxiety and depression in MSD, while concurrently improving core symptoms of MSD, such as PE and ED. Notably, CHM formulations demonstrated superior efficacy over SSRIs in improving both psychological scales, including Self-Rating Anxiety Scale [MD = -9.11, 95% CI (-11.53, -6.70), *P* < .05], Self-Rating Depression Scale [MD = -9.85, 95% CI (-14.07, -5.63), *P* < .05], the Hamilton Depression Rating Scale (HAMD) [MD = -5.30, 95% CI (-11.61, 1.01), *P* > .05], and the Hamilton Anxiety Rating Scale [MD = -3.89, 95% CI (-4.52,-3.27), *P* < .05], as well as MSD-specific metrics, such as International Index of Erectile Function-5 [MD = 3.26, 95% CI (1.98, 4.53), *P* < .05] and intravaginal ejaculation latency time [MD = 1.60, 95% CI (0.82, 2.37), *P* < .05]. Importantly, the lack of statistical significance in HAMD scores in our analysis may be attributed to the differences in treatment responses between the PE and ED populations.

**Clinical Implications:**

It provides evidence-based support to address the limitations of separating physical and mental symptoms in traditional treatment, further substantiates its application value within the integrated medical model, and explores new research avenues for designing personalized treatment plans for patients.

**Strengths and Limitations:**

A first-of-its-kind systematic evaluation was conducted to assess the comprehensive efficacy of traditional CHM in alleviating anxiety and depression symptoms while improving sexual function indicators among patients with MSD. The limited number of studies constitutes the most significant limitation.

**Conclusions:**

Our findings provide the first robust evidence that CHM significantly alleviates anxiety and depression in MSD patients, while concurrently improving core MSD symptoms, such as PE and ED.

## Introduction

Male Sexual Dysfunction (MSD) encompasses a range of conditions that impair male sexual function, including erectile dysfunction (ED), premature ejaculation (PE), and hypoactive sexual desire disorder (HSDD).^1^ The prevalence of MSD increases with age; ~50% of men over the age of 40 experience varying degrees of sexual dysfunction.^2^ In studies from certain countries, the prevalence of ED can be as high as 79.4%, and in specific populations, such as patients with diabetes or cardiovascular diseases, the prevalence of ED is even higher, reaching 90% or more.^3^^,^^4^ The overall incidence of PE is estimated to be between 20% and 30%, with 36% to 63% classified as primary PE and 16% to 28% as secondary PE.^5-7^ ED and PE can each serve as a risk factor for the other, a condition that has been defined as loss of control over erection and ejaculation.^8^ Beyond its impact on physical health, MSD significantly affects mental well-being and interpersonal relationships, potentially exacerbating psychological issues such as anxiety and depression.^9^ The pathophysiological mechanisms underlying MSD are multifaceted, encompassing neurological, vascular, endocrine, and psychological factors. Metabolic disorders, including dyslipidemia, hypertension, and hyperglycemia, as well as other chronic conditions, may lead to microcirculatory impairments and abnormal neural conduction, consequently affecting erectile and ejaculatory functions.^10^ Endocrine dysregulation in patients with diabetes not only impairs testosterone synthesis but may also exacerbate ED by compromising vascular endothelial function.^11^ Research indicates that the prevalence of sexual dysfunction in individuals with anxiety disorders is significantly higher compared to the general population, and elevated anxiety levels are strongly associated with reduced sexual satisfaction.^12^ Furthermore, lifestyle factors such as smoking, excessive alcohol consumption, and physical inactivity can also adversely affect sexual function.^13^ Research indicates that sexual dysfunction is closely associated with anxiety and depression, forming a complex interrelationship that often traps patients in a vicious cycle. Anxiety and depression not only exacerbate the symptoms of sexual dysfunction but can also lead to avoidance of sexual activities, thereby further increasing the psychological burden on patients.^14^^,^^15^ There is a significant association between depression and sexual dysfunction, particularly among male patients, where the prevalence of depression can reach up to 60% or higher.^16^^,^^17^ Therefore, it is imperative to accord increased attention to anxiety and depression in the context of MSD.

Currently, the treatment options for MSD primarily include pharmacotherapy, psychological counseling, and lifestyle modifications. In drug therapy, phosphodiesterase-5 inhibitors are the first-line agents for treating ED, while selective serotonin reuptake inhibitors (SSRIs) are primarily used for managing PE.^18^ The use of SSRIs is off-label in many countries, with the only exception of Dapoxetine, which is not available worldwide. SSRIs can have negative effects on sexual function, by suppressing sexual desire, reducing erectile function, and delaying ejaculatory latency times (in those without PE).^19^^,^^20^ However, while these medications can improve MSD symptoms to some extent, their efficacy in addressing concomitant psychological issues such as anxiety and depression is limited.^21^ Additionally, they may cause side effects including headache, flushing, and gastrointestinal discomfort.^22^ Studies indicate that ~30% to 40% of patients exhibit either a suboptimal response or no response at all to these medications.^23^ Against this backdrop, Chinese Herbal Medicine (CHM) have increasingly garnered attention. Studies have demonstrated their efficacy in improving sexual function and alleviating anxiety and depression, with relatively fewer side effects compared to conventional treatments.^11^

CHM has demonstrated significant advantages in treating anxiety and depression among patients with MSD, particularly in improving psychological well-being, enhancing sexual function, and providing robust clinical research evidence. The multi-component and multi-target nature of CHM confers a unique advantage in treating complex diseases, as its ability to simultaneously act on multiple pathological mechanisms enhances therapeutic efficacy.^15^^,^^24^ Studies have shown that Yinyanghuo (Epimedium) can enhance sexual desire and improve erectile function by regulating neurotransmitter balance, while also effectively alleviating symptoms of anxiety and depression.^11^^,^^25^ CHM prescriptions, such as Bazhen Decoction and Sijunzi Decoction, demonstrate significant efficacy in regulating qi and blood and enhancing physical constitution, while also effectively alleviating symptoms of anxiety and depression, thereby contributing to improved sexual function.^12^^,^^26^ However, although individual studies have explored the effects of CHM on MSD and associated psychological conditions including anxiety and depression, the research findings remain inconsistent. Particularly in cases involving comorbidities between MSD and mental health disorders such as anxiety and depression, a comprehensive evaluation of the therapeutic efficacy of CHM remains to be established.

This meta-analysis addressed the existing knowledge gap regarding CHM in managing MSD with comorbid anxiety and depression. By comprehensively synthesizing clinical evidence, we evaluated CHM's therapeutic efficacy and established an evidence-based framework for future clinical trials, thereby informing standardized treatment protocols for this complex comorbidity.

## Methods

This study adhered to the Preferred Reporting Items for Systematic Reviews and Meta-Analyses (PRISMA) guidelines.^27^^,^^28^ The study protocol was registered on PROSPERO (CRD420250652254; https://www.crd.york.ac.uk/prospero/).

### Inclusion and exclusion criteria

#### Type of study

Only RCTs were included. However, the studies must be in either English or Chinese.

#### Type of participants

Adult male patients (18-65 years) diagnosed with MSD and comorbid anxiety and/or depression.

#### Type of intervention

The experimental group must receive treatment with either single-agent or combination CHM, while the control group may receive placebo, conventional pharmacotherapy, or psychotherapy. All included trials strictly defined the experimental intervention as CHM monotherapy, which refers to the exclusive use of Chinese herbal formulations (eg, decoctions, granules, or capsules) without concurrent acupuncture, moxibustion, or manual therapies.

#### Type of outcome measures

Outcome measures required at least one assessment of anxiety, depression, or the core symptoms of MSD. Primary outcomes included the Self-Rating Anxiety Scale (SAS),^29^ the Self-Rating Depression Scale (SDS),^30^ the Hamilton Anxiety Rating Scale (HAMA),^31^ and the Hamilton Depression Rating Scale (HAMD).^32^ Secondary outcomes comprised intravaginal ejaculation latency time (IELT) scores and International Index of Erectile Function-5 (IIEF-5) scores.^33^^,^^34^ It is worth noting that IELT and IIEF-5 are high-benefit scores where a higher value represents improvement. Conversely, SAS, SDS, HAMA, and HAMD are low-benefit scores where a lower value indicates improvement.

#### Inclusion criteria

To be eligible for inclusion in the study, participants were required to meet the diagnostic criteria for PE and ED as outlined in the “Chinese Guidelines and Expert Consensus on Diagnosis and Treatment of Male Genitourinary Diseases (2016 Edition)”.^33^ These disorders were prioritized due to their high comorbidities with depression/anxiety. Other conditions (eg, HSDD) were excluded due to insufficient herbal intervention studies meeting inclusion criteria. Specifically, participants were required to have an IELT of less than 1 minute or an IIEF-5 score of 21 or lower.

#### Exclusion criteria

Observational studies, cross-sectional studies, animal studies, and other non-RCTs were excluded.

### Search strategy

Two investigators (Z.X. and J.L.) independently and comprehensively searched four Chinese databases (China National Knowledge Infrastructure, Wanfang Data, VIP Information Resource Platform, and China Biology Medicine) and four international databases (PubMed, Web of Science, EMBASE, Cochrane Library). The search covered all records published from the inception of each database to 24 February 2025, with no geographical or language restrictions. All databases were systematically interrogated using Boolean logic operators and appropriate truncation techniques to ensure exhaustive identification of relevant studies. The detailed search strategy is presented in eIndex 1 of the supplementary material.

### Study selection and data extraction

Two researchers (Z.X. and J.L.) independently screened titles and abstracts to confirm eligibility based on inclusion criteria, and subsequently conducted full-text evaluations of all selected studies. The following data were extracted using standardized data extraction templates: first author's name; publication year; participant age range; sample size; intervention protocols in experimental and control groups; treatment duration; primary outcome measures; and documented adverse events. In cases of discrepancies between reviewers, a senior researcher (X.Z.) was consulted, and a consensus was reached through structured deliberation.

### Risk of bias assessment

Two researchers (Z.X. and J.L.) independently assessed the risk of bias for each included RCT using the Cochrane Risk of Bias tool (RoB 2.0).^35^ The evaluation covered five core domains: random sequence generation, allocation concealment, blinding of participants and investigators, blinding of outcome assessors, and completeness of outcome data. Studies were categorized as having high, low, or unclear risk of bias based on predefined evaluation criteria. In cases of discrepancies between assessors, a senior researcher (X.Z.) was consulted to facilitate consensus resolution through structured deliberation.

### Statistical analysis

Meta-analysis was performed using Stata 16.0 and Review Manager (RevMan) version 5.3. Given that each score represented continuous variables with varying measurement scales, score differences between pre-treatment and post-treatment measurements were systematically extracted. The standardized mean difference (SMD) with 95% confidence intervals (95% CIs) was selected as the primary effect measure. Heterogeneity was assessed through Cochran's Q test (α = 0.10) complemented by I^2^ quantification. For studies demonstrating acceptable heterogeneity (I^2^ < 50%), a fixed-effects model was implemented. When substantial heterogeneity was identified (I^2^ ≥ 50%), a random-effects model was applied with subsequent subgroup analyses conducted to elucidate heterogeneity sources. Where quantitative synthesis proved methodologically inappropriate, a narrative synthesis was performed. For trials reporting only pre-post values, mean changes were calculated by subtracting baseline measurements from post-intervention values, with corresponding standard deviations (SD) derived using the Cochrane Handbook-recommended formula.

### Subgroup analyses

Subgroup analyses were conducted to investigate sources of heterogeneity when significant variation was detected. Prespecified stratification factors included publication year, intervention modality, treatment duration, and disease classification. For subgroups with sufficient data, mixed-effects meta-regression was performed to examine treatment effect modifiers. For subgroups with insufficient data for quantitative synthesis, a systematic qualitative synthesis was conducted, which involved within-study comparisons, cross-study consistency evaluations, and evidence quality assessments using the GRADE framework, thereby enabling meaningful interpretation of findings without formal meta-analytic integration.^36^

### Sensitivity analysis

When significant heterogeneity persisted following subgroup analyses, sensitivity analyses were conducted to assess result stability. The sensitivity analysis protocol comprised iterative re-running of meta-analytic models with sequential exclusion of studies meeting high risk of bias criteria (score ≥ 4 on a modified Newcastle-Ottawa Scale) and statistical outliers identified via Galbraith plot diagnostics. Comparative effect size estimation between primary and sensitivity analyses was performed using Hartung-Knapp adjustment, with between-analysis discrepancies quantified through random-effects variance decomposition. This methodological framework enabled comprehensive assessment of result robustness while elucidating heterogeneity contributors through differential exclusion effects on pooled estimates.

### Publication bias

When the meta-analysis included 10 or more studies, publication bias was evaluated using Egger's regression test. The statistical findings were graphically supplemented with funnel plots incorporating precision estimates to assess potential small-study effects in effect size distribution asymmetry.

## Results

### Study identification and selection

**Figure 1:**
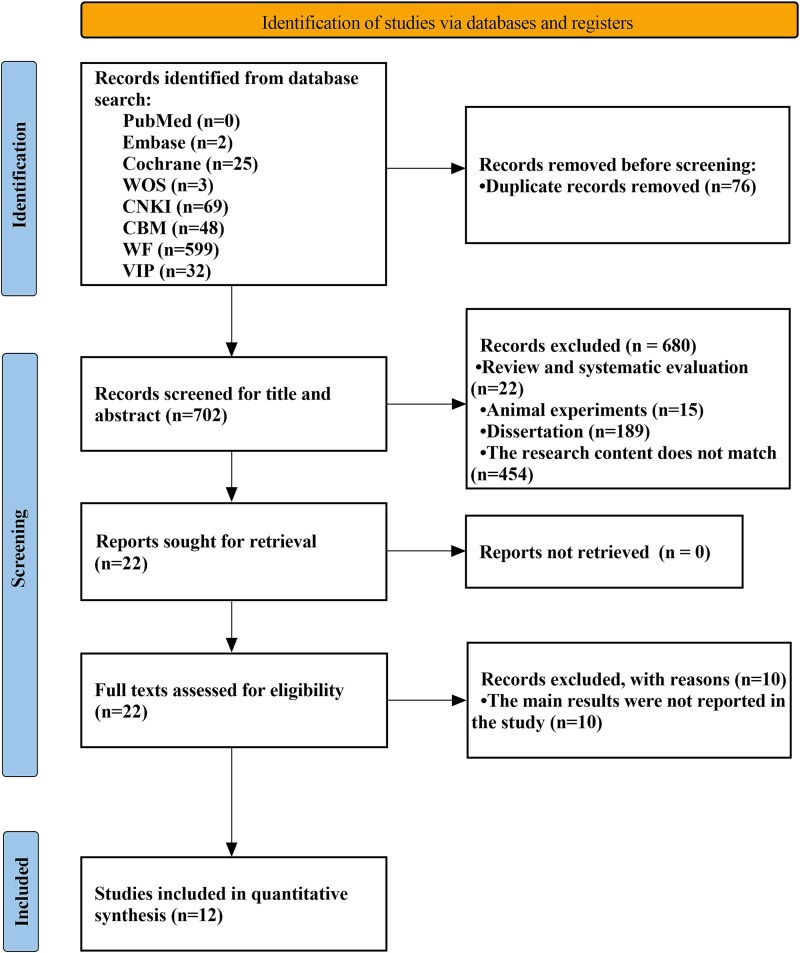
Flow chart showing results of the literature search and study inclusion. The process followed the PRISMA guidelines. PRISMA = preferred reporting items for systematic reviews and meta-analyses.

A total of 778 studies were retrieved. After removing duplicates and following strict inclusion and exclusion criteria for screening, 12 studies were eventually included in our analysis. The detailed procedures for each step are illustrated in Figure 1.

### Study characteristics

Our review included a total of 12 studies, with a cumulative sample size of 1050 participants. All studies were published in Chinese and conducted in China. A total of 519 participants were assigned to the control group, receiving either placebo, psychotherapy, or SSRIs; 531 participants were allocated to the treatment group and received interventions involving CHM alone or in combination with SSRIs. Regarding primary outcome indicators, six studies reported depression scores using the SAS; four studies reported scores on the SDS; four studies reported scores on the HAMD; and two studies reported scores on the HAMA. In terms of secondary outcome measures, five studies reported IIEF-5 scores, while five studies reported IELT scores. The baseline characteristics of participants across these studies are summarized in Table 1.

**Table 1:** Baseline characteristics of the included studies.

References	Country	Experimental			Control			Course	Type of MSD	Type of outcomes
		Treatment	n	Age (Mean + sd)	Treatment	n	Age (Mean + sd)			
Chen WT 2022^41^	China	Shugan Jieyu Prescription	45	31.12 ± 2.46	Sildenafil Citrate	43	31.23 ± 2.18	8 W	ED	IIEF-5 SAS
Jiang LJ 2013^42^	China	oral Zhenwei decoction+External Zhuangyang San	49	32.64 ± 5.25	Trazodone Hydrochloride	49	34.68 ± 6.75	30D	ED	IIEF-5 HAMD HAMA
Li ZQ 2020 ^43^	China	ultra-powder Second Gufang Yiyuan Decoction	100	43.25 ± 7.86	Zhibai Dihuang Pills	100	42.31 ± 7.77	3 M	ED	IIEF-5 SDS
Li ZM 2014^44^	China	Chaihu plus Longgu Muli Decoction	30	-	Tadalafil	30	-	20D	ED	HAMD
Liang YL 2017^45^	China	Shugan Yiyang Capsule	40	33.15 ± 6.20	Sertraline Hydrochloride Tablets	40	33.37 ± 6.50	6 W	PE	IELT HAMD
Liu LT 2021^46^	China	Xinnao Shutong Capsule	38	-	Sildenafil Citrate	38	-	8 W	ED	SAS SDS
Liu WD 2024^47^	China	Chonghuang Bu Shen Capsule	60	25.47 ± 3.80	Suo Yang Bu Shen Capsules	60	24.85 ± 3.40	2 W	PE	IELT HAMA
Ma G 2017^48^	China	Shugan Yi Yang Capsules	33	34.0 ± 2.2	Sertraline Hydrochloride Tablets	33	33.2 ± 2.1	4 W	PE	IELT HAMD
Ma ZY 2025^49^	China	Chaihu Shugan San Capsules	20	30.50 ± 5.89	Placebo capsules	20	30.30 ± 3.17	4 W	ED	IIEF-5 SAS SDS
Su W 2023^50^	China	Compound Xuanju Capsules	40	42.14 ± 5.71	Sildenafil Citrate	40	41.84 ± 5.08	3 M	ED	IIEF-5 SAS SDS
Xie FX 2015^51^	China	Chaihu Longgu Oyster soup	40	36.3 ± 5.4	Sertraline Hydrochloride Tablets	30	37.0 ± 5.8	4 W	PE	IELT SAS
Yu C 2016^52^	China	Yangxin Shugan Decoction	36	27.18 ± 8.27	Behavioral Psychotherapy	36	26.51 ± 8.99	2 M	PE	IELT SAS

M, Moon; W, week; D, day; SAS, the Self-Rating Anxiety Scale; SDS, the Self-Rating Depression Scale; HAMD, the Hamilton Depression Rating Scale; HAMA, the Hamilton Anxiety Rating Scale; IIEF-5, International Index of Erectile Function-5; IELT, intravaginal ejaculation latency time.

### Risk of bias

**Figure 2:**
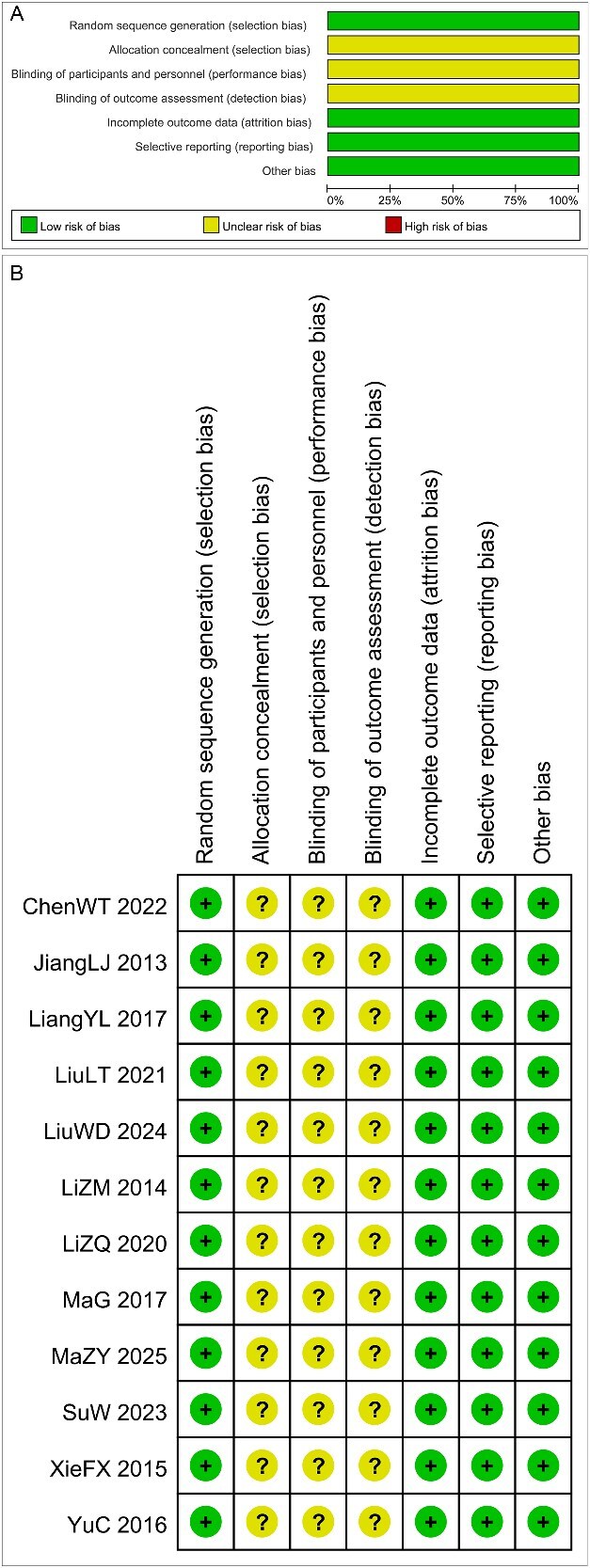
Risk of bias assessment. Green indicates low risk of bias, yellow indicates medium risk of bias, and red indicates high risk of bias.

All studies described the methods used for randomization but did not clarify whether the assigned sequences were adequately concealed. Consequently, these studies were judged to have an unclear risk of selection bias. None of the studies explicitly detailed the implementation of blinding procedures, leading to their classification as having an unclear risk of performance or detection bias. All included studies reported complete outcome data and were therefore considered to have a low risk of reporting bias. All studies presented their pre-specified outcomes, with no indication of selective reporting identified. The overall methodological quality of the trials was assessed as having a moderate risk of bias. The results of the risk-of-bias assessment are summarized in Figure 2.

### Meta-analysis

#### The SAS scores

**Figure 3:**
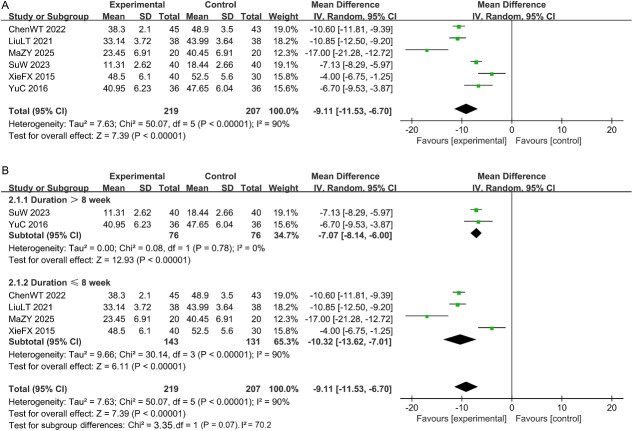
(A) Forest plot of studies for the SAS scores. (B) Subgroup analysis for the SAS scores.

Six studies reported the SAS scores. The analysis revealed substantial statistical heterogeneity for this outcome [I^2^ = 90% > 50%, *P* < .1; Figure 3A]. Consequently, a random-effects model was employed for the analysis. The results indicated that the treatment group significantly reduced the SAS scores in MSD patients compared with the control group [MD = -9.11, 95% CI (-11.53, -6.70), *P* < .05]. To further investigate the potential reasons for the higher heterogeneity, we conducted a subgroup analysis based on the course of treatment. The results indicated that the differences between groups did not reach statistical significance. Subgroup heterogeneity suggested that there might be clinically significant differences in effect sizes among different subgroups, which could be influenced by unmeasured confounding variables or the limitations of subgroup sample sizes. See Figure 3B.

#### The SDS scores

**Figure 4:**
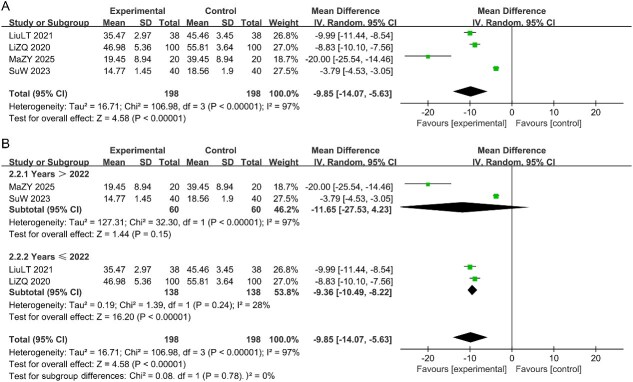
(A) Forest plot of studies for the SDS scores; (B) Subgroup analysis for the SDS scores.

Four studies reported the SDS scores, followed by a meta-analysis to assess the efficacy of CHM. The meta-analysis, conducted using a random-effects model, revealed the significant superiority of the CHM group compared with the control group [MD = -9.85, 95% CI (-14.07, -5.63), *P* < .05, I^2^ = 97%; Figure 4A], indicating substantial heterogeneity despite the significant differences. To further explore the potential sources of heterogeneity, we conducted a detailed subgroup analysis stratified by the publication year of the included studies. The results indicate that there is satisfactory homogeneity between the two groups, suggesting that the statistical process in the combined analysis of each study is robust and reliable. See Figure 4B.

#### The HAMD scores

**Figure 5:**
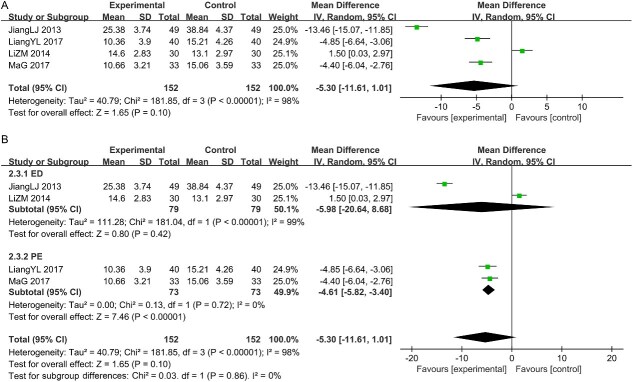
(A) Forest plot of studies for the HAMD scores. (B) Subgroup analysis for the HAMD scores.

A total of four studies reported the HAMD scores. The heterogeneity test revealed substantial heterogeneity, indicating that a random-effects model was appropriate for the meta-analysis [I^2^ = 98% > 50%, *P* < .05; Figure 5A]. However, the analysis using the random-effects model showed that the CHM group did not significantly reduce the HAMD scores compared with the control group [MD = -5.30, 95% CI (-11.61, 1.01), *P* > .05]. To further explore the potential sources of heterogeneity, we performed subgroup analyses stratified by disease classification. The results indicated that the PE group exhibited excellent homogeneity and achieved statistical significance; in contrast, the ED group displayed substantial heterogeneity and no statistical significance. See Figure 5B.

#### The HAMA scores

**Figure 6:**
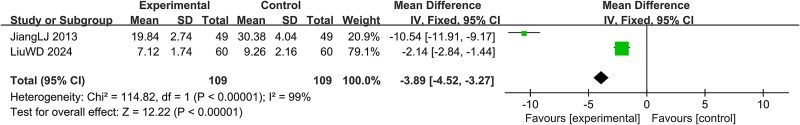
Forest plot of studies for the HAMA scores.

Two studies provided the HAMA scores. To contrast the efficacy of CHM with the control group, a random-effect meta-analysis was performed [I^2^ = 99% > 50%, *P* < 0.1; Figure 6]. Despite the high level of heterogeneity observed in the results, the efficacy of CHM was significantly greater than that of the control group, with the difference achieving statistical significance [MD = -3.89, 95% CI (-4.52,-3.27), *P* < .05]. Owing to the limited number of available relevant RCTs, it was not feasible to perform subgroup analysis.

#### The IIEF-5 scores

**Figure 7:**
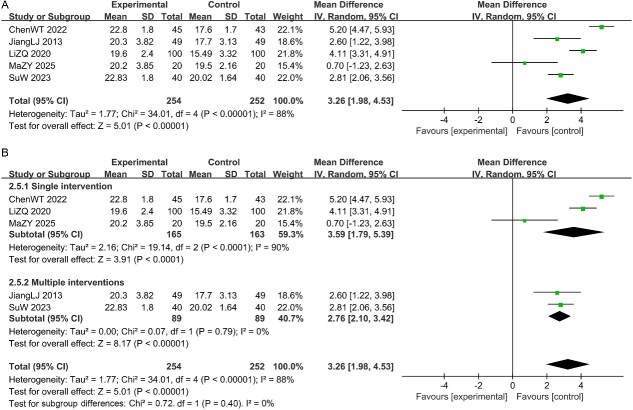
(A) Forest plot of studies for the IIEF-5 scores; (B) Subgroup analysis for the IIEF-5 scores.

Five studies reported the IIEF-5 scores. Random-effects model analysis (I^2^ = 88% > 50%, *P* < .1; Figure 7A) indicated that the CHM group had significantly higher IIEF-5 scores compared to the control group [MD = 3.26, 95% CI (1.98, 4.53), *P* < .05]. Subgroup analysis based on intervention measures revealed acceptable homogeneity among the groups, suggesting that the statistical process in the pooled analysis of the included studies was robust and reliable. See Figure 7B.

#### The IELT scores

**Figure 8:**
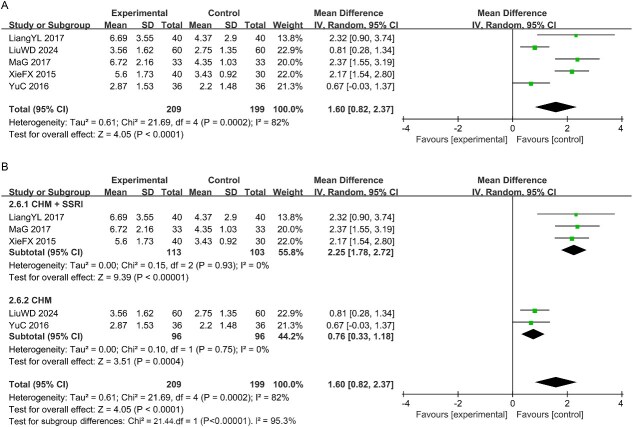
(A) Forest plot of studies for the IELT scores; (B) Subgroup analysis for the IELT scores.

Five studies documented IELT scores. Statistical analysis revealed considerable heterogeneity for this outcome [I^2^ = 82% > 50%, *P* < .1; Figure 8A], prompting the application of a random-effects model. The CHM group exhibited a significant reduction in IELT scores among MSD patients compared to controls [MD = 1.60, 95% CI (0.82, 2.37), *P* < .05]. To investigate potential sources of elevated heterogeneity, subgroup analyses stratified by intervention measures were conducted. These analyses demonstrated inter-group homogeneity, thereby confirming the robustness of the statistical process in the pooled analysis. See Figure 8B.

### Sensitivity analysis

Sensitivity analyses of all studies revealed no significant changes, thereby confirming the robustness and reliability of the results. The additional details are presented in eIndex 2 of the supplementary material.

### Publication bias

As recommended by the Cochrane Handbook, caution is advised against using funnel plot asymmetry testing when fewer than 10 studies are included in a meta-analysis.^37^ With such a small sample size, the statistical power of the test is insufficient to reliably differentiate between random variation and true asymmetry.^38^ Consequently, we did not construct a funnel plot for this analysis.

### Adverse

Seven studies reported adverse events. Of these, two studies reported that no adverse events were experienced by the patients during the intervention. Adverse events observed in the remaining five studies are summarized in Table 2. Specifically, adverse events in the CHM group included gastrointestinal discomfort, headache, dizziness, fatigue and weakness, nausea and vomiting, nasal congestion, and disgust. In the control group, adverse events comprised facial edema, gastrointestinal discomfort, headache, dizziness, decreased libido, nausea and vomiting, nasal congestion, and disgust.

**Table 2:** Adverse events.

References	Experimental		Control	
	Adverse events	n	Adverse events	n
Chen WT 2022	-	-	Facial edema	2/43
Li ZQ 2020	Gastrointestinal discomfort	2/100	-	-
Liang YL 2017	Gastrointestinal discomfort	1/40	Gastrointestinal discomfort	2/40
	Dizziness	1/40	Dizziness	1/40
	Feeling exhausted and weak	2/40	Decreased libido	3/40
Liu WD 2024	-	-	-	-
Ma G 2017	Dizziness	1/29	Dizziness	1/29
	Nausea and vomiting	1/29	Nausea and vomiting	2/29
	-	-	Decreased libido	1/29
Su W 2023	Headache	3/40	Headache	2/40
	Nasal congestion	1/40	Nasal congestion	1/40
	Disgusting	2/40	Disgusting	1/40
Xie FX 2015	-	-	-	-

## Discussion

This study presents the first systematic meta-analysis to evaluate the efficacy and safety of CHM for managing anxiety and depression symptoms in patients with MSD. By synthesizing data from 12 RCTs involving 1050 participants, our findings provide the first robust evidence that CHM significantly alleviates anxiety and depression in MSD, while concurrently improving core symptoms of MSD, such as PE and ED. Notably, CHM formulations demonstrated superior efficacy over SSRIs in improving both psychological scales (SAS, SDS, HAMA, HAMD) and MSD-specific metrics (IELT, IIEF-5). Subgroup analysis of the HAMD score revealed that the substantial heterogeneity primarily originated from differences in treatment responses between PE and ED populations. Specifically, while HAMA and SDS concentrate on affective dimensions that are closely associated with MSD-related distress, HAMD places greater emphasis on somatic symptoms, which are less pertinent to this specific population. Subgroup analysis revealed differential effects between the PE and ED subgroups, potentially reflecting treatment responses specific to distinct pathophysiological mechanisms.

High heterogeneity observed across outcome measures underscores the complexity of CHM interventions. Our subgroup analyses suggest that treatment duration (SAS), publication year (SDS), intervention protocol (IELT, IIEF-5), and disease classification (HAMD) may contribute to variability. The persistent high heterogeneity in SAS subgroup analysis despite stratification by treatment time implies additional unmeasured confounders, such as differences in herbal formulation standardization or psychometric assessment protocols across studies. These findings stress the need for rigorous reporting standards in CHM trials, including detailed descriptions of herbal compositions and quality control measures. Notably, our analysis identified discrepancies in measurement outcomes across assessment instruments, potentially attributable to differential tool orientations. Specifically, patient-reported measures including SAS and SDS capture comprehensive biopsychosocial manifestations, whereas clinician-rated scales such as HAMA and HAMD focus predominantly on quantifiable clinical parameters. This methodological divergence provides a plausible explanation for outcome variability in our meta-analysis and indicates that Chinese Herbal Medicine may exert dual therapeutic effects—enhancing subjective symptom awareness while ameliorating targeted pathophysiological mechanisms in male sexual dysfunction populations. Subgrouping by publication year revealed declining effect sizes, potentially reflecting improved methodological rigor in recent trials reducing placebo effects.

CHM exerts therapeutic effects on MSD with comorbid anxiety and depression through multi-target interventions involving neurotransmitter regulation, metabolic modulation, and neuroendocrine-immune integration. CHM formulations restore serotonin (5-HT) and norepinephrine balance by inhibiting monoamine oxidase activity and upregulating synaptic neurotransmitter reuptake. For instance, Chaihu Shugan San, which contains Saikosaponins, has been shown to significantly improve HAMD scores via this mechanism.^39-41^ Herbs such as *Gardenia jasminoides*, which contain iridoids, suppress corticosterone overproduction and downregulate glucocorticoid receptor expression in hippocampal neurons, thereby reversing stress-induced dysregulation of the HPA axis.^42^ Saikosaponins, the primary active components of Bupleurum scorzonerifolium Willd, have demonstrated significant improvement in depression-like behavior, attenuation of central inflammation, and marked inhibition of neuronal pyroptosis in mice across both in vivo and in vitro models.^43^ CHM formulas suppress hyperactivity of the hypothalamic–pituitary–adrenal (HPA) axis and reduce proinflammatory cytokines by modulating glucocorticoid receptor expression. For instance, iridoids from *G. jasminoides* exert anti-inflammatory effects through NF-κB pathway inhibition, thereby restoring neuroendocrine-immune homeostasis.^44^ Tianmeng Oral Liquid alleviate depressive symptoms by modulating nucleotide, energy, and amino acid metabolism, thereby replenishing ATP and reducing oxidative stress in MSD-related neuronal circuits.^45^ The multicomponent nature of CHM enables it to simultaneously target neurotransmitter imbalance, inflammation, and vascular dysfunction, thereby distinguishing it from conventional single-pathway antidepressants.^44^^,^^46^

Our research results indicate that CHM can effectively alleviate anxiety and depression in MSD patients, with a relatively low incidence of adverse reactions. However, there were some limitations to this systematic review and meta-analysis. Firstly, we developed rigorous inclusion criteria to ensure the quality of the studies, but this may result in a limited number of studies. Secondly, all the included studies were from China, which may lead to regional bias in the results. Lastly, significant heterogeneity was observed due to variations in the quality of the included studies. To address these limitations, we conducted a subgroup analysis stratified by publication date, treatment duration, intervention measures, and disease type. Unfortunately, some subgroup analyses failed to identify the above factors as potential sources of heterogeneity. Given that CHM treatment involves complex formulation variables, we speculate that the main sources of heterogeneity may stem from CHM preparation itself, particularly in herb combinations, treatment duration, and standardization of decoction processes. Therefore, standardized CHM treatment protocols should be developed in future studies to reduce heterogeneity, enhance research quality, and evaluate cross-cultural applicability through multinational trials.

## Conclusion

Our findings provide the first robust evidence that CHM significantly alleviates anxiety and depression in MSD patients, while concurrently improving core MSD symptoms, such as PE and ED. However, our proposal is constrained by the limited number of RCTs included, which are also of low quality. We expect multi-center and high-quality RCTs to compensate for the deficiencies in this study and further prove this conclusion.

## Supplementary Material

eIndex_1The_detailed_search_strategy_qfaf048

eIndex_2Sensitivity_analysis_qfaf048

PRISMA_2020_checklist_qfaf048
