# Supplementary material for: Efficacy of Chinese herbal medicine in the treatment of anxiety and depression in male sexual dysfunction: a systematic review and meta-analysis
Source: Sex Med. 2025 Jul 10;13(3):qfaf048. doi: 10.1093/sexmed/qfaf048 (PMC12240729; doi:10.1093/sexmed/qfaf048)
Supplement: eIndex_1The_detailed_search_strategy_qfaf048 [file eindex_1the_detailed_search_strategy_qfaf048.docx]

Supplementary Material

**Detailed Literature Search Strategy**

**Appendix 1** Search Strategy

| Database | Search strategy | amount |
| --- | --- | --- |
| **PubMed** | | |
| #1 | Search: ("Premature Ejaculation"[Mesh]) OR ("Erectile Dysfunction"[Mesh]) | 22555 |
| #2 | Search: ((((((male sexual dysfunction[Title/Abstract]) OR (Dysfunction, Erectile[Title/Abstract])) OR (Impotence[Title/Abstract]))) OR (Male Impotence[Title/Abstract])) OR (Ejaculation, Premature[Title/Abstract])) OR (Premature Ejaculations[Title/Abstract]) | 7123 |
| #3 | #1 OR #2 | 25547 |
| #4 | Search: ("Anxiety"[Mesh]) OR ("Depression"[Mesh]) | 248018 |
| #5 | Search: (((Nervousness[Title/Abstract]) OR (Anxiousness[Title/Abstract])) OR (Depressive Symptom[Title/Abstract])) OR (Depression, Emotional[Title/Abstract]) | 7220 |
| #6 | #4 OR #5 | 252171 |
| #7 | Search: "Medicine, Chinese Traditional"[Mesh] | 26255 |
| #8 | Search: (chinese herbal medicine[Title/Abstract]) OR (herbal[Title/Abstract]) | 56097 |
| #9 | #7 OR #8 | 79220 |
| #10 | Search: (randomized controlled trial[Publication Type] OR randomized[Title/Abstract] OR placebo[Title/Abstract]) | 107216 |
| #11 | #3 AND #6 AND #9 AND #10 | 0 |

| **Database** | **Search strategy** | **amount** |
| --- | --- | --- |
| **Embase** | | |
| #1 | ‘erectile dysfunction’:ab,ti OR ‘premature ejaculation’:ab,ti OR ‘male sexual dysfunction’:ab,ti OR ‘Dysfunction, Erectile’:ab,ti OR ‘Impotence’:ab,ti OR ‘Male Impotence’:ab,ti OR ‘Ejaculation, Premature’:ab,ti OR ‘Premature Ejaculations’:ab,ti | 43451 |
| #2 | ‘anxiety’:ab,ti OR ‘depression’:ab,ti OR ‘Nervousness’:ab,ti OR ‘Anxiousness’:ab,ti OR ‘Depressive Symptom’:ab,ti OR ‘Depression, Emotional’:ab,ti | 869615 |
| #3 | ‘chinese herbal medicine’:ab,ti OR ‘traditional chinese medicine’:ab,ti OR ‘herbal’:ab,ti | 123224 |
| #4 | ‘randomized controlled trial’:ab,ti OR ‘randomized’:ab,ti OR ‘placebo’:ab,ti OR ‘RCT’:ab,ti | 1277410 |
| #5 | #1 AND #2 AND #3 AND #4 | 2 |

| **Database** | **Search strategy** | **amount** |
| --- | --- | --- |
| **Web of Science** | | |
| #1 | TS=(erectile dysfunction OR premature ejaculation OR male sexual dysfunction OR Dysfunction, Erectile OR Impotence OR Male Impotence OR Ejaculation, Premature OR Premature Ejaculations) | 35912 |
| #2 | TS=(anxiety OR depression OR Nervousness OR Anxiousness OR Depressive Symptom OR Depression, Emotional) | 925411 |
| #3 | TS=(chinese herbal medicine OR traditional chinese medicine OR herbal) | 111841 |
| #4 | TS=(randomized controlled trial OR randomized OR placebo OR RCT) | 1232476 |
| #5 | #1 AND #2 AND #3 AND #4 | 3 |

| Database | Search strategy | amount |
| --- | --- | --- |
| **Cochrane** | | |
| #1 | (erectile dysfunction):ab,ti,kw OR (premature ejaculation):ab,ti,kw OR (male sexual dysfunction):ab,ti,kw OR (Dysfunction, Erectile):ab,ti,kw OR (Impotence):ab,ti,kw OR (Male Impotence):ab,ti,kw OR (Ejaculation, Premature):ab,ti,kw OR (Premature Ejaculations):ab,ti,kw | 6212 |
| #2 | (anxiety):ab,ti,kw OR (depression):ab,ti,kw OR (Nervousness):ab,ti,kw OR (Anxiousness):ab,ti,kw OR (Depressive Symptom):ab,ti,kw OR (Depression, Emotional):ab,ti,kw | 150592 |
| #3 | (chinese herbal medicine):ab,ti,kw OR (traditional chinese medicine):ab,ti,kw OR (herbal):ab,ti,kw | 22518 |
| #4 | (randomized controlled trial):ab,ti,kw OR (Randomized):ab,ti,kw OR (placebo):ab,ti,kw OR (RCT):ab,ti,kw | 1332684 |
| #5 | #1 AND #2 AND #3 | 25 |

| Database | Search strategy | amount |
| --- | --- | --- |
| CNKI | | |
| #1 | 男性性功能障碍 + 性功能障碍 + 阳痿 + 勃起障碍 + 勃起功能障碍 + 性障碍 + 早泄 | 23400 |
| #2 | 焦虑 + 抑郁 | 306800 |
| #3 | 中草药 + 中药 + 中医 + 中成药 + 胶囊 + 方 + 丸 + 汤 + 散 | 2006900 |
| #4 | 随机对照 + [随机对照试验](javascript:toDetail('cross','D016449','110');) + 随机对照实验 + 随机对照研究 + RCT + 随机对照 + 随机 | 2586400 |
| #5 | #1 AND #2 AND #3 AND #4 | 69 |

| Database | Search strategy | amount |
| --- | --- | --- |
| WF | | |
| #1 | 男性性功能障碍 OR 性功能障碍 OR 阳痿 OR 勃起障碍 OR 勃起功能障碍 OR 性障碍 OR 早泄 | 41936 |
| #2 | 焦虑 OR 抑郁 | 534595 |
| #3 | 中草药 OR 中药 OR 中医 OR 中成药 OR 胶囊 OR 方 OR 丸 OR 汤 OR 散 | 41784270 |
| #4 | [随机对照试验](javascript:toDetail('cross','D016449','110');) OR 随机对照实验 OR 随机对照研究 OR RCT OR 随机对照 OR 随机 | 3454091 |
| #5 | #1 AND #2 AND #3 AND #4 | 599 |

| Database | Search strategy | amount |
| --- | --- | --- |
| CBM | | |
| #1 | ("性障碍"[常用字段:智能] OR "勃起障碍"[常用字段:智能] OR "男性性功能障碍"[常用字段:智能]) OR (("早泄"[不加权:扩展]) OR ("勃起功能障碍"[不加权:扩展]) OR ("阳萎"[不加权:扩展]) OR ("性功能障碍, 生理性"[不加权:扩展] OR "性功能障碍, 心理性"[不加权:扩展])) | 58451 |
| #2 | ("抑郁"[不加权:扩展]) OR ("焦虑"[不加权:扩展]) | 93517 |
| #3 | ("中药"[常用字段:智能] OR "中医"[常用字段:智能] OR "中成药"[常用字段:智能] OR "胶囊"[常用字段:智能] OR "方"[常用字段:智能] OR "丸"[常用字段:智能] OR "汤"[常用字段:智能] OR "散"[常用字段:智能]) OR ("中草药"[不加权:扩展]) | 8961022 |
| #4 | ("随机对照实验"[常用字段:智能] OR "随机对照研究"[常用字段:智能] OR "RCT"[常用字段:智能] OR "随机对照"[常用字段:智能] OR "随机对照"[常用字段:智能]) OR ("随机对照试验"[不加权:扩展]) | 239008 |
| #5 | #1 AND #2 AND #3 | 48 |

| Database | Search strategy | amount |
| --- | --- | --- |
| VIP | | |
| #1 | 男性性功能障碍 + 性功能障碍 + 阳痿 + 勃起障碍 + 勃起功能障碍 + 性障碍 + 早泄 | 24168 |
| #2 | 焦虑 + 抑郁 | 173462 |
| #3 | 中草药 + 中药 + 中医 + 中成药 + 胶囊 + 方 + 丸 + 汤 + 散 | 1326667 |
| #4 | 随机对照 + [随机对照试验](javascript:toDetail('cross','D016449','110');) + 随机对照实验 + 随机对照研究 + RCT + 随机对照 + 随机 | 2514370 |
| #5 | #1 AND #2 AND #3 AND #4 | 32 |
