# Supplementary material for: Efficacy of Chinese herbal medicine in the treatment of anxiety and depression in male sexual dysfunction: a systematic review and meta-analysis
Source: Sex Med. 2025 Jul 10;13(3):qfaf048. doi: 10.1093/sexmed/qfaf048 (PMC12240729; doi:10.1093/sexmed/qfaf048)
Supplement: eIndex_2Sensitivity_analysis_qfaf048 [file eindex_2sensitivity_analysis_qfaf048.docx]

Supplementary Material


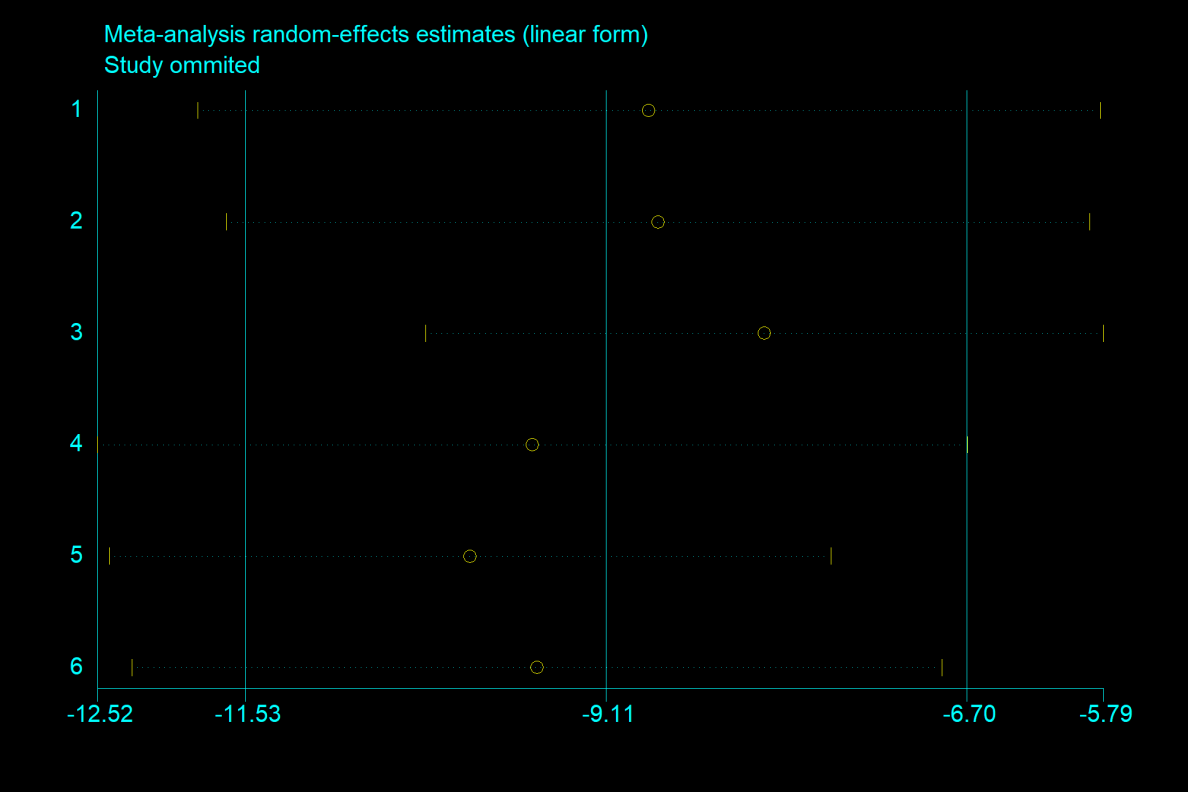


Sensitivity analysis chart (SAS)


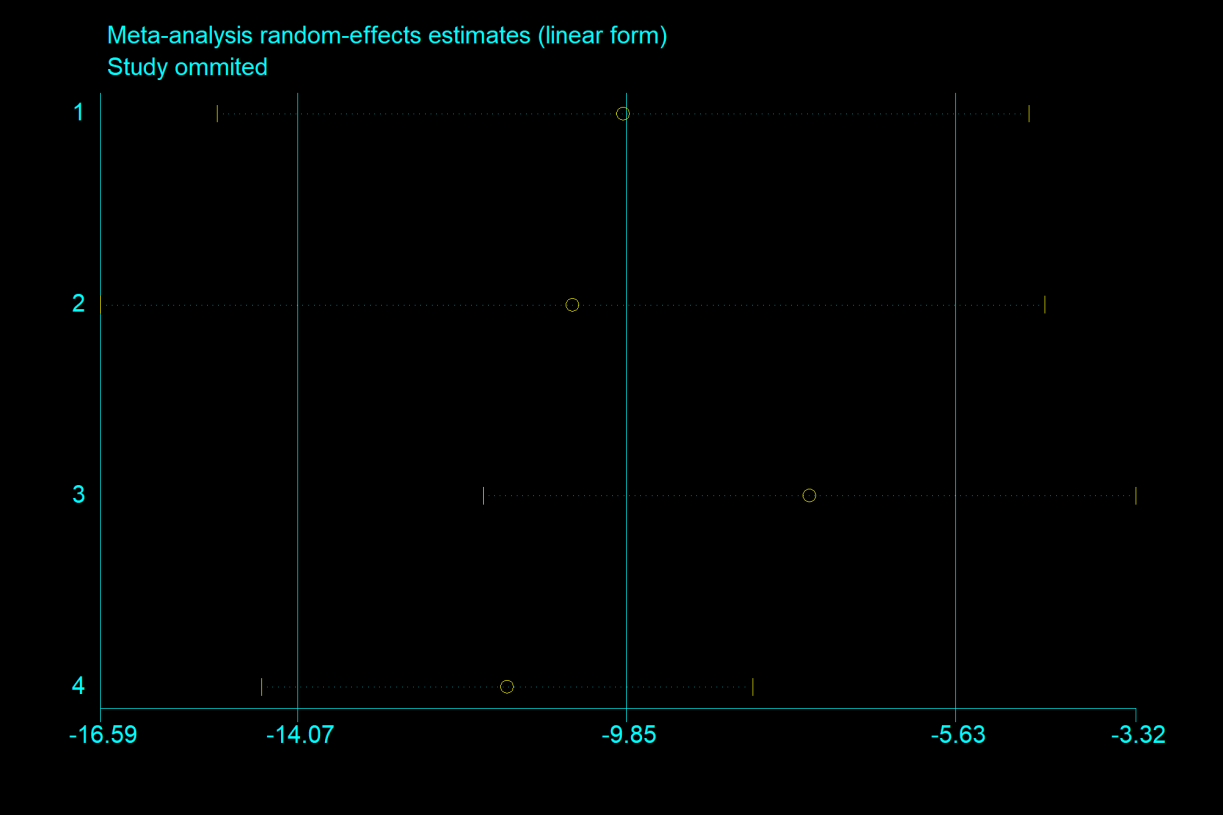


Sensitivity analysis chart (SDS)


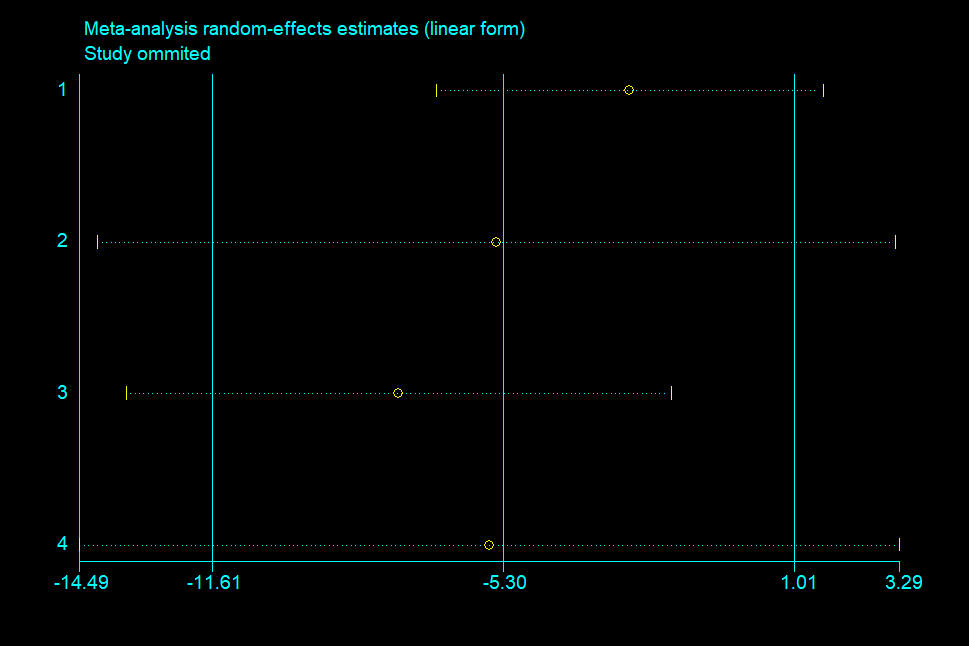


Sensitivity analysis chart (HAMD)


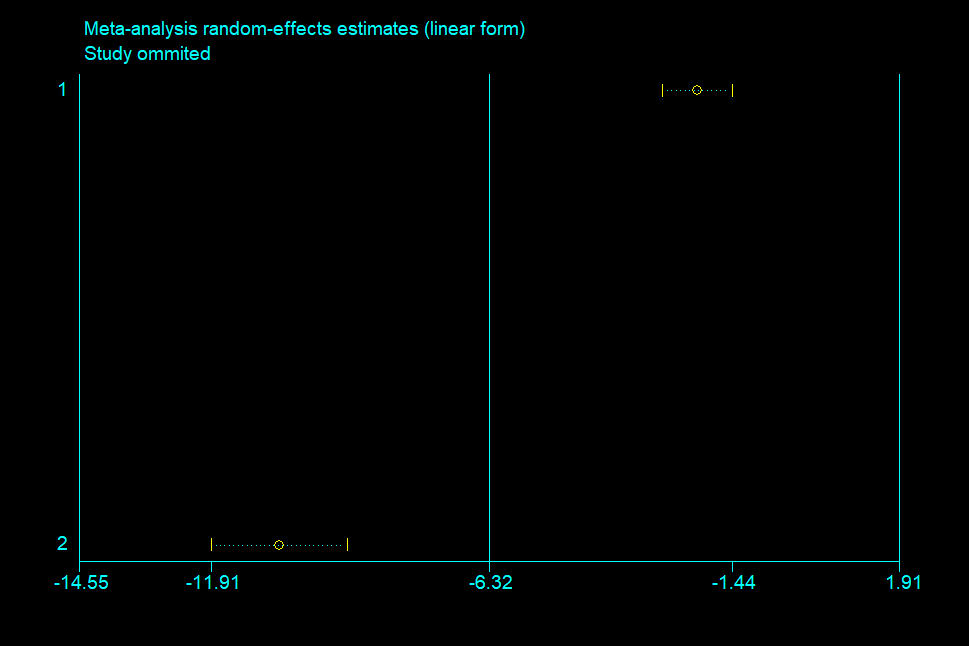


Sensitivity analysis chart (HAMA)


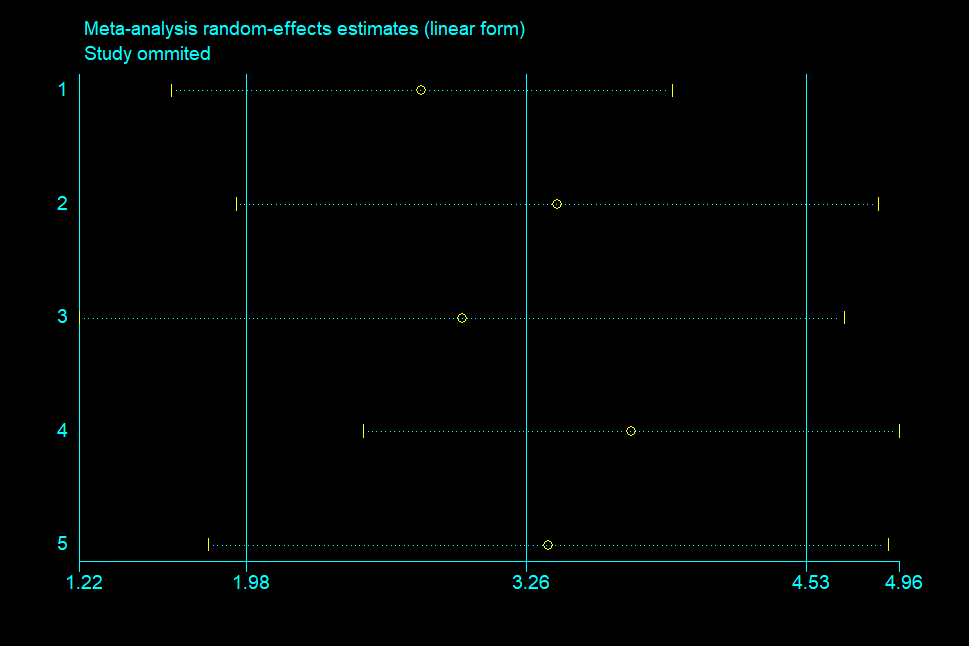


Sensitivity analysis chart (IIEF-5)


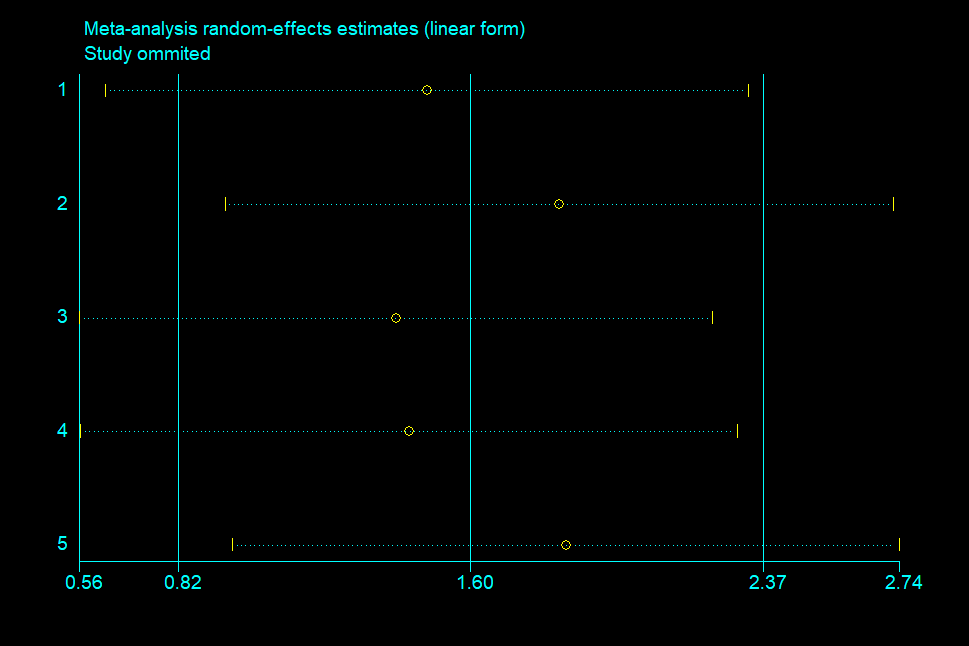


Sensitivity analysis chart (IELT)
